# Celiac Disease Diagnosed in an Older Adult Patient with a Complex Neuropsychiatric Involvement: A Case Report and Review of the Literature

**DOI:** 10.3390/brainsci10070426

**Published:** 2020-07-03

**Authors:** Emma Falato, Fioravante Capone, Federico Ranieri, Lucia Florio, Marzia Corbetto, Chiara Taffon, Cinzia Niolu, Giorgio Di Lorenzo, Vincenzo Di Lazzaro

**Affiliations:** 1 Unit of Neurology, Neurophysiology, Neurobiology, Department of Medicine, Università Campus Bio-Medico di Roma, via Álvaro del Portillo 200, 00128 Rome, Italy; e.falato@unicampus.it (E.F.); f.capone@unicampus.it (F.C.); 2 Department of Neuroscience, Biomedicine and Movement Sciences, University of Verona, 37134 Verona, Italy; federico.ranieri@univr.it; 3 Unit of Neurology, IRCCS Casa Sollievo della Sofferenza, San Giovanni Rotondo, 71013 Foggia, Italy; luciaflorio81@gmail.com; 4 Department of Neurology, Santa Maria Goretti Hospital, 04100 Latina, Italy; m.corbetto@ausl.latina.it; 5 Unit of Pathology, Università Campus Bio-Medico di Roma, via Álvaro del Portillo 200, 00128 Rome, Italy; c.taffon@unicampus.it; 6 Unit of Psychiatry, Department of Systems Medicine, University of Rome Tor Vergata, via Montpellier 1, 00133 Rome, Italy; niolu@med.uniroma2.it (C.N.); di.lorenzo@med.uniroma2.it (G.D.L.)

**Keywords:** celiac disease, spastic paraparesis, delusional jealousy

## Abstract

We present a case of celiac disease (CD) diagnosis in a 75-year-old woman with a long-term history of chronic delusional jealousy and a complex neurological involvement. The case describes a very unusual clinical picture, provides some clinical clues, and highlights the importance of being aware of CD extraintestinal manifestations in order to get a timely diagnosis.

## 1. Introduction

Celiac disease (CD) is a chronic, multi-organ autoimmune disease that affects the small bowel in genetically predisposed individuals, triggered by gluten ingestion and treated with a gluten-free diet (GFD) [1,2,3]. The prevalence of CD is rising [1], as well as the incidence of CD cases presenting with extraintestinal symptoms [4] and in adulthood [5]. Gastrointestinal manifestations of CD include diarrhea, abdominal bloating, weight loss, and nutrient malabsorption [3]. A set of extraintestinal manifestations have been associated with CD among which are iron-deficiency anemia, abnormalities in liver function tests, bone disease (osteopenia/osteoporosis), skin disorders, fatigue, and a wide spectrum of neurological and psychiatric disorders [1,3,5,6,7,8,9]. Neurologic manifestations may either precede or follow CD diagnosis and are thought to occur in about 10–20% of patients with established CD [1,10]. The occurrence of psychiatric symptoms in CD patients has also been known for a long time [11,12] and is increasingly reported [13,14]. Many of the neuropsychiatric disorders associated with CD have been linked to cross-reacting immune responses or to nutritional deficiencies [6]. However, the pathophysiology of CD-associated neuropsychiatric manifestations remains largely elusive, and further systematic studies are needed to address the mechanisms of the underlying nervous system pathology. Since GFD showed to improve neuropsychiatric manifestations associated with CD in some patients [1,7,13,15,16,17,18], clinicians’ awareness about CD extraintestinal manifestations should be high.

## 2. Case Report

A 75-year-old Caucasian woman came to our neurology outpatient clinic for a 10-year history of progressive walking disorder, which started at the age of 65 and slowly worsened. She complained of “stiffness” and “heaviness” in her legs, frequent falls, and painful muscle cramps. More recently, for about two years, she had noticed a slowing of her movements. She stated that for all these symptoms she had already undergone clinical and blood tests, genetic and neuroimaging assessments without receiving a diagnosis, and that she had only been treated with conventional physiotherapy.

Importantly, the patient had received a diagnosis of chronic delusional jealousy at the age of 64, a few months after an acute episode of psychosis. For this, she had been treated with haloperidol (unknown dosage) for about one year, and then with aripiprazole (2.5 mg daily) during the last nine years. Other comorbidities were high blood pressure, osteoporosis, and early insomnia.

Her past medical history was unremarkable. Her father died of a stroke, her mother of breast cancer; she had one son with great vessel transposition and no other relevant diseases in her family health history. She denied a family history of psychiatric disorders. Her medication list was aripiprazole 2.5 mg daily, acetylsalicylic acid 100 mg daily, ramipril 2.5 mg, amiloride-hydrochlorothiazide 2.5–25 mg alt.d., and delorazepam 0.25 mg daily. Furthermore, she added that she was occasionally taking natural supplements for a few years for mild bloating, not otherwise specified, and that she had recently supplemented vitamin B12. She had no allergies, and no history of alcohol, tobacco, and drug use. She had a body mass index of 24 kg/m^2^.

At our neurological examination, we observed a complex neurological involvement. Spastic paraparesis was the prominent feature and was associated with extrapyramidal, cerebellar, and neuropathic signs. Indeed, we observed scanning speech, hypomimia, bradykinesia, intermittent right resting tremor and bilateral postural tremor, positive finger-to-nose test, spastic paraparesis with moderate distal weakness, and lower limbs hyperreflexia with a bilateral Babinski sign. She had an unsteady and wide-based gait, with reduced arm swing. She was well-oriented and scored 30/30 on the Mini-Mental State Examination (MMSE) [19]. She had no dysphagia, no nystagmus, no bladder dysfunction, and no autonomic failure.

Blood tests revealed mild normocytic anemia (hemoglobin: 11.7 g/dL normal values (n.v.) 12–16); mild hypoproteinemia (total protein 4.90 g/dL, n.v. 6.20–8.10); mild hypoalbuminemia (3.04 g/dL; n.v. 3.20–4.20); severe folate deficiency (folic acid 0.70 ng/mL; normal values (n.v.) >5.4). Vitamin B12 and serum iron were at the lower level of the normal range. Ferritin was within the normal range.

**Figure 1:**
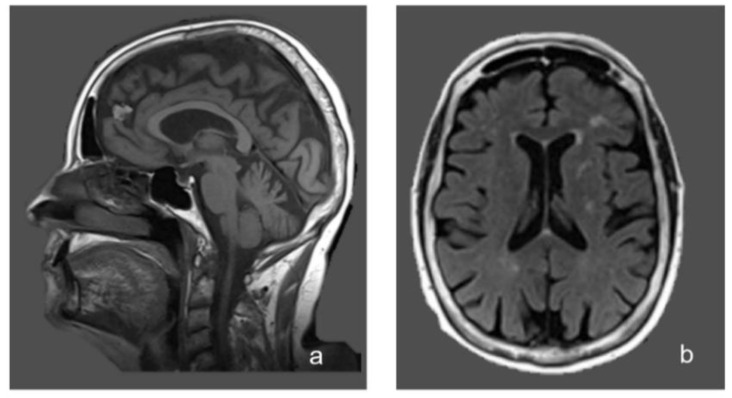
Sagittal (**a**) and axial (**b**) magnetic resonance imaging (MRI) Fluid-Attenuated Inversion Recovery (FLAIR) images of the patient, showing multiple areas of white matter hyperintensity and cerebellar atrophy.

At the instrumental assessments:

- nerve conduction studies and electromyography (NCSs/EMG) documented a motor axonal polyneuropathy with signs of active denervation at the lower limbs;
- somatosensory and motor potentials (SEPs and MEPs) documented a complete absence of responses from the lower limbs;
- brain magnetic resonance imaging (MRI) showed non-specific periventricular gliosis (Figure 1);
- spinal cord MRI was normal.

Furthermore, the patient had already undergone dopamine transporter (DaT) single-photon emission tomography (SPECT) brain imaging with Ioflupane I-123 injection at the age of 73, which had detected slight striatal hypoperfusion bilaterally.

Psychiatric consultation confirmed the diagnosis of chronic delusional jealousy according to the Diagnostic and Statistical Manual of Mental Disorders (DSM)-5 criteria [20] and the adequacy of the ongoing therapy with aripiprazole 2.5 mg daily. The patient was accessible to the psychiatric interview, lucid and well-oriented; when her husband was present, she manifested an increased level of tension with eye contact avoidance, hand-wringing, and incongruous laugh. Delusions of jealousy were still present, albeit partially criticized and associated with a lower emotional component compared to the onset, when she was not on aripiprazole. From the interview, it also emerged that, in the last years, the patient had developed a progressive tendency to social withdrawal. However, no significant levels of apathy, depression, and anxiety were recorded.

**Figure 2:**
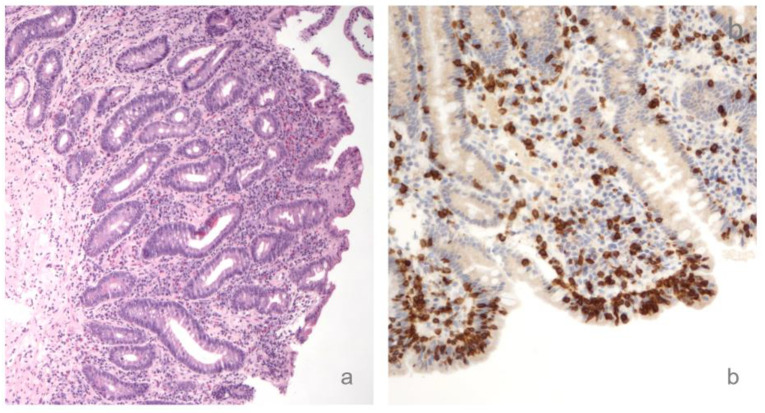
(**a**) A fragment of the patient’s duodenal mucosa showing villous atrophy, glandular crypt hyperplasia, low enterocytes height, irregular brush-border and cytoplasmic vacuoles; (**b**) CD3 immunohistochemical staining of the duodenal mucosa showing a pathological increase of intraepithelial lymphocytes.

Due to the finding of severe folic acid deficiency, and despite the patient reporting no clear gastrointestinal symptoms except for mild and occasional bloating, a blood screening for causes of malabsorption was performed, which ended in finding high serological levels of anti-transglutaminase (TTG) and anti-endomysial (EMA) antibodies (TTG: 36.3 AU/mL, normal values: 0.2–8; EMA: present 1:10, normal value: absent—assessed through TTG and EMA IgA ELISA kit. Anti-gliadin and vitamin E dosages not available). The diagnosis of celiac disease stage 3c (according to Marsh–Oberhuber classification) was then confirmed through a duodenal biopsy (Figure 2).

The patient’s colonoscopy was normal, except for the distal ileum, where the mucosa showed a typical appearance of nodular lymphoid hyperplasia, without signs of acute inflammation.

GFD was started, in association with short-term folic acid replacement. At the two-month follow-up, the normalization of serum TTG and EMA antibodies and of folate levels was observed. Serum homocysteine was normal, and folic acid replacement was discontinued. During the following months, the patient noticed a subjective improvement in her gait, reduced leg stiffness and fatigability, and she had no further episodes of falling. At our objective follow-up evaluations during the following two years, her walking impairment, which had previously been progressive, did not further deteriorate. As an objective measure, at the nine-month follow-up, we found visible lumbar (N22) and cortical (N40) responses at the lower limb SEPs, which had previously been absent, with increased latency. MEPs remained persistently absent at lower limbs. In addition, her psychiatric condition remained stable, with no further episodes of acute psychosis. The patient’s adherence to GFD was assessed during our two-year follow-up through dietary review and serial measurements of vitamins and of TTG and EMA antibodies levels [1,21]. No follow-up duodenal biopsy was performed.

A written informed consent for publication was obtained from the patient.

## 3. Discussion

We described the case of a woman who had an insidious onset of neuropsychiatric symptoms (chronic delusional jealousy and progressive walking impairment due to complex neurological involvement) at the age of 64, and was diagnosed with CD after 11 years, through serologic (TGG and EMA antibody positivity) and histologic confirmation. The detection of a severe folic acid deficiency on blood tests raised the suspicion of malabsorption and guided the clinical reasoning to the research of CD as a possible unifying cause of the patient’s complex neuropsychiatric picture. 

CD can manifest with or be accompanied by many neurological and psychiatric disorders, both in children and in adults [1,7,22,23]. The first descriptions of a link between CD and neurological and psychiatric disorders date back long ago [11,12,24]. Over the years, an increasing number of case reports, case series, and population-based studies strongly supported the link between CD and neuropsychiatric disorders [9,15,25,26,27,28,29,30], although a few studies have yielded conflicting results [31,32,33].

Psychiatric disorders commonly observed in untreated CD patients include mood and anxiety disorders, attention deficit, autism, and schizophrenia [13]. A recent systematic review and meta-analysis confirmed that CD is associated with an increased risk of autistic spectrum disorder, attention deficit hyperactivity disorder, depression, anxiety, and eating disorders [14]. A significant positive association between CD and psychosis has also been recently demonstrated [34]. Neurologic diseases frequently observed in patients with CD and gluten-related disorders [2] include cerebellar ataxia, peripheral neuropathy, headache, and white matter abnormalities. Less commonly, epilepsy, myopathy, myelopathy, acute inflammatory demyelinating polyradiculoneuropathy, restless legs syndrome, dystonia, myoclonus, stiff person syndrome, cognitive impairment, pseudotumor cerebri, and brain calcifications have been described [1,7,10,16]. Very recently, an analysis of data from the UK Biobank, including 104 CD patients with a mean age of 63 years and 195 matched controls, further highlighted that subjects with CD have higher rates of cognitive deficit, worse mental health, and white matter changes, supporting the association between celiac disease and neurologic and psychological features [35].

The mechanisms linking CD and neuropsychiatric manifestations remain largely elusive. Current evidence suggests that neuropsychiatric manifestations of CD are immune-mediated (cross-reacting antibodies, immune-complex deposition, T cell cytotoxicity), although gluten-related toxicity, microbiome, vitamin deficiencies, and genetic association could also play a role [1,15,16,36,37]. Further, neuroinflammation triggered by autoimmune disorders and by gut microbiota alterations is being increasingly proposed as a possible link between CD and some neuropsychiatric disorders [38,39,40,41], and represents a future area of interest for research.

The association between delusional jealousy (DJ) and CD, found in our patient, has never been described before, to our best knowledge.

Delusional jealousy (also known as morbid/pathological/paranoid jealousy; Othello syndrome; delusional disorder-jealous type; conjugal paranoia) is a psychiatric disorder characterized by the firm and false belief that a spouse or lover is unfaithful [42]. DJ is classified among the subtypes of the delusional disorder in the DSM-5 [20]. DJ has been described in functional and organic psychosis, including dementia, stroke, alcoholism, traumatic brain injury (TBI) and in parkinsonian patients [43,44,45,46,47,48], but never in association with CD. In a retrospective case series of 105 patients affected by delusional jealousy, the average onset was in the 6th decade, and the condition was often associated with neurologic disorders [48]. The neural bases of DJ should still be clarified. Some studies highlighted the role of frontal lobes [48], and a model on the feeling of jealousy and its delusional form has been proposed, in which dopaminergic frontostriatal circuits, the ventromedial prefrontal cortex, the insula, and their related functions of reward, mentalizing, and self-related processing have been included [42]. In this case, the patient had no history of alcohol abuse, TBI, stroke, or dementia, and developed the psychiatric symptoms several years before the extrapyramidal symptoms. 

The neurological picture found in our patient is also unique for its multisystemic involvement.

Indeed, the patient’s walking impairment and neurological symptoms were linked to the involvement of all the following: pyramidal pathways at the spinal cord level (spastic paraparesis, bilateral Babinski sign); cerebellum (gait ataxia, scanning speech, positive finger-to-nose test); peripheral nerves (axonal motor neuropathy, distal weakness); and extrapyramidal system (hypomimia, bradykinesia, intermittent right resting tremor, reduced arm swing).

Ataxia [49], peripheral neuropathies [50], as well as brain white matter lesions [51] have been described in many cases of CD with neurological manifestations. However, other findings such as spastic paraparesis with normal spine MRI [52,53], myelopathic signs with normal spine MRI and SEPs improvement after GFD [54], parkinsonism and psychosis in CD [55], and parkinsonian symptoms improvement after GFD [56] have been described only anecdotally and not in combinations like in this case.

We performed a literature search in PubMed using the following terms: “(celiac disease OR coeliac disease) AND (psychiatric) AND (neurologic or neurological OR neuropsychiatric OR neuro-psychiatric)”. We looked for case reports and/or case series and/or population-based studies, in English language, already published in the literature, which included, similarly to our case, adult patients (aged 18 or more), with biopsy-proven CD, who presented both neurological and psychiatric symptoms. Of the 68 articles found with these search keywords, and after the consultation of 24 more articles found from their references lists, only five case reports were found that responded to these inclusion characteristics (Table 1). From this search, the need for a more standardized and systematic reporting (inclusive of CD diagnosis criteria, antibody status, order of presentation of the neuropsychiatric symptoms with respect to CD diagnosis, effects of GFD) emerged.

Furthermore, we found one cross-sectional study on biopsy-proven adult (aged 18 years or more) CD patients with “neurological and/or psychiatric conditions”. About 35% of the 72 patients (mean age 51 + 15) reported a history of psychiatric problems, while the most common neurological conditions recorded were migraine 28%, carpal tunnel syndrome 20%, vestibular dysfunction 8%, seizures 6%, and myelitis 3% [62].

The clinical picture described in our patient shares some similarities with the gluten ataxia phenotype [49] (insidious onset in adulthood, dysarthria, gait ataxia, absence of prominent gastrointestinal symptoms, cerebellar atrophy, and white matter abnormalities on MRI), frequently observed in non-celiac gluten sensitivity (NCGS) [63]. However, our patient also had spastic paraparesis, peripheral neuropathy, extrapyramidal signs, and chronic delusional jealousy. Other differential diagnoses excluded, not fully explaining the patient’s clinical picture, were spinocerebellar ataxia and multiple system atrophy. Our patient had no autonomic failure, no nystagmus, no dysphagia, and no cognitive dysfunction.

The cornerstone of CD treatment is the dietary exclusion of gluten [1,5]. Several studies demonstrated that GFD could prevent neuropsychiatric manifestations of CD and improve these manifestations in some patients [1,7,13,15,16,17,18,64,65]. Further, functional data derived from transcranial magnetic stimulation studies suggested that a long-lasting GFD could partially restore the imbalance between intracortical excitatory and inhibitory circuits in CD patients [66]. Instead, prolonged gluten exposure has been associated with poor prognosis after GFD [7,49]. All these data highlight the importance of a timely diagnosis for CD patients. After GFD, our patient had a subjective improvement, and we objectively observed the stabilization of the previously progressive neurologic impairment and the improvement of SEPs. The persistence of absent lower limb MEPs could have been influenced by the patient’s motor axonal neuropathy. Regarding the patient’s extrapyramidal signs, it has to be taken into account that the chronic treatment with aripiprazole, considered mandatory and thus maintained, might have contributed to the parkinsonian symptoms and to the limited gait improvement after GFD [67]. Aripiprazole is an antipsychotic drug with an uncertain mechanism of action. In animal models, it showed both, agonist or antagonist presynaptic D2 receptors activity, depending on the dopaminergic tone [68]. Among its proposed mechanisms of action, besides partial agonism on D2 dopaminergic receptors and antagonist action on 5HT_2_A serotoninergic receptors, a partial agonism on 5HT_1_A serotoninergic receptors has been proposed, which theoretically reduces motor side effects [69]. Furthermore, the hypothesis of drug-induced parkinsonism is improbable at the dosage assumed by the patient. In addition, the asymmetrical resting tremor and the DaT-SPECT, which suggested a defect at the presynaptic dopaminergic receptors level, are less in favor of drug-induced parkinsonism [70].

The incidence of CD cases presenting in adulthood and with minimal or no gastrointestinal symptoms is rising [4,5]. Such a paradigm shift requires high clinician awareness. It has been estimated that CD diagnosis can have a median delay of 3.5 years when there are no gastrointestinal complaints (e.g., diarrhea, malabsorption, weight loss, and gassy distension) [71]. In the case described here, the advanced age of the patient, the absence of clear gastrointestinal symptoms, and the complex clinical picture made the diagnosis challenging. Bloating was described as very mild and sporadic and did not represent a prominent clinical clue. Instead, osteoporosis, mild anemia and hypoproteinemia can be considered systemic CD manifestations. Importantly, the severe folic acid deficiency represented a key finding and a “red flag” for possible malabsorption, which guided us towards the final diagnosis.

## 4. Conclusions

The case described highlights the importance of considering CD in the differential diagnosis of unexplained neuropsychiatric disorders, even in older adults and even in the absence of gastrointestinal symptoms.

## Figures and Tables

**Table 1:** Case reports about adult (age >18) patients, with biopsy-proven celiac disease (CD), with neurological and psychiatric disorders.

Reference	Arshad et al. 2018 [57]	Ravindra et al. 2008 [58]	Ryan et al. 2007 and 2009 [59,60]	Gonzalez Aleman et al. 2006 [55]	Dseplat-Jego et al. 2003 [61]
**Age at CD diagnosis**	22	27	55	37	76
**Gender**	M	F	M	F	F
**CD diagnosis**	Duodenal biopsy	Duodenal biopsy EMA	Small bowel biopsy TTG	Duodenal biopsy	Small bowel biopsy; TTG, EMA, and anti-gliadin antibodies
**Extraintestinal manifestations**	Microcytic hypochromic anemia	Microcytic hypochromic anemia, cheilitis	None	None	Macrocytic anemia, low serum albumin, vitamin B12 deficiency, cachectic status
**Neurological signs and symptoms**	Gradual cognitive impairment, speech difficulties for 2 years, brisk lower limb reflexes, increased tone in all four limbs	Myotonic dystrophy	Rapidly progressive neurological decline: headache, seizures, ataxia, myoclonus, cognitive impairment	Parkinsonism	Mental confusion, auditory and visual hallucinations, memory impairment
**Psychiatric symptoms**	Aggressive behavior, not otherwise specified	Irritability	Anxiety	Psychosis with severe anxiety and marked psychological agitation	Depressive state, withdrawal attitude, paranoid delirium
**Neuroimaging**	Normal	N.A.	Brain MRI: leukoencephalopathy, pulvinar sign	Brain MRI: periventricular white matter hyperintensities. Tanscranial ultrasound: enlarged echogenic areas in the substantia nigra bilaterally	N.A.
**Treatment**	GFD	GFD, iron, calcium and folic acid supplements for 8 weeks	Probably already on GFD (CD diagnosis 6 months before), anticonvulsants, immunosuppressive treatment	Already on GFD (not specified for how long), clozapine	GFD, Vit B12 supplementation
**Effect of treatment on neuropsychiatric symptoms**	Improvement	None	None	Clozapine improved psychiatric symptoms. Parkinsonism did not change after clozapine discontinuation	Improvement
